# The universal algebra of the electromagnetic field III. Static charges and emergence of gauge fields

**DOI:** 10.1007/s11005-022-01515-4

**Published:** 2022-03-21

**Authors:** Detlev Buchholz, Fabio Ciolli, Giuseppe Ruzzi, Ezio Vasselli

**Affiliations:** 1 Mathematisches Institut, Universität Göttingen, Bunsenstr. 3-5, 37073 Göttingen, Germany; 2 Dipartimento di Matematica, Universitá di Roma “Tor Vergata”, Via della Ricerca Scientifica 1, 00133 Roma, Italy

**Keywords:** Electromagnetic field, Static charges, Gauge fields, C*-algebras, 81T05, 83C47, 57T15

## Abstract

A universal C*-algebra of gauge invariant operators is presented, describing the electromagnetic field as well as operations creating pairs of static electric charges having opposite signs. Making use of Gauss’ law, it is shown that the string-localized operators, which necessarily connect the charges, induce outer automorphisms of the algebra of the electromagnetic field. Thus they carry additional degrees of freedom which cannot be created by the field. It reveals the fact that gauge invariant operators encode information about the presence of non-observable gauge fields underlying the theory. Using the Gupta-Bleuler formalism, concrete implementations of the outer automorphisms by exponential functions of the gauge fields are presented. These fields also appear in unitary operators inducing the time translations in the resulting representations of the universal algebra.

## Introduction

We construct in this article an extension of the universal C*-algebra of the electromagnetic field in Minkowski space, presented in [3]. It includes, in addition to the fields, operators creating pairs of static electric charges with opposite signs. The primary purpose of our analysis is the demonstration that such pairs are inevitably accompanied by non-observable gauge fields. This generalizes results in our previous article [5], based on a kinematical framework and relying on canonical commutation relations of the fields. The upshot of our present investigation is the insight that, quite generally, the observable operators encode information about the existence of non-observable gauge fields, underlying the theory. In other words, the presence of unobservable gauge fields is traceable by physical effects.

Our restriction to static (infinitely heavy) charges greatly simplifies the analysis since such charges can sharply be localized; the localization properties of dynamical charges with finite masses are more fuzzy [6]. Yet the appearance of gauge fields is not related to this idealization. It is solely a consequence of Gauss’ law, which does not depend on the mass of a system carrying an electric charge.

Extending arguments in [3], we will construct a universal C*-algebra, which in addition to the electromagnetic field contains operators describing pairs of opposite charges, localized at spacelike separated points. The crucial additional input encoded in this algebra is Gauss’ law according to which the values of the individual charges can be determined by measurements of the electric flux around their respective positions. The resulting algebra is shown to describe a local, Poincaré invariant net on Minkowski space, describing the electromagnetic effects induced by static charges.

The principal point in our investigation consists of the demonstration that the string-localized operators, connecting the charges, define outer automorphisms of the subalgebra of the electromagnetic field. This will be established by different means, firstly in the abstract framework and then by exhibiting a representation of the algebra, based on the Gupta-Bleuler formalism and an abelian algebra of operators creating static charges. Within the present general setting our results corroborate the claim made in [3] that gauge fields cannot be replaced by elaborate limits of the electromagnetic field.

Our article is organized as follows. In the subsequent section we establish our notation and recall the definition of the universal algebra ${{\mathfrak {V}}}$ of the electromagnetic field. We will also exhibit outer automorphisms of this algebra which can be interpreted as *gauge bridges* between static charges. In Sect. 3 we extend the algebra ${{\mathfrak {V}}}$ to an algebra ${{\mathfrak {W}}}$ containing unitaries, inducing these automorphisms. For the sake of gauge invariance, we add to it an abelian algebra of unitary operators which generate the static charges. In Sect. 4 we construct physically significant representations of the electromagnetic part of the resulting algebra. The article closes with brief conclusions.

## The universal algebra

In this section we recall the definition and basic properties of the universal C*-algebra ${{\mathfrak {V}}}$ of the electromagnetic field [3], show how local charge measurements are described, and exhibit outer automorphisms which modify the charges.

The unitary elements of the algebra, describing exponentials of the *intrinsic* vector potential $A_I$, are labeled by real, vector-valued test functions *g* with compact support and vanishing divergence, $\delta g \doteq \partial _\mu g^\mu = 0$; they form a real vector space, denoted by ${{\mathcal {C}}}_1({{\mathbb {R}}}^4)$. The relation between $A_I$ and the electromagnetic field *F* is given by $e^{i a A_I(g)} = e^{i a F(f)}$. Here $a \in {{\mathbb {R}}}$ and *g* is any solution of the equation $g = \delta f$ for given real, skew-tensor-valued test function $f \in {{\mathcal {D}}}_2({{\mathbb {R}}}^4)$, where $\delta : {{\mathcal {D}}}_2({{\mathbb {R}}}^4) \rightarrow {{\mathcal {C}}}_1({{\mathbb {R}}}^4)$ is defined by $(\delta f)^\mu \doteq - 2 \partial _\nu f^{\mu \nu }$. Such solutions exist according to Poincaré’s Lemma, and the potential $A_I$ is unambiguously defined in view of the homogeneous Maxwell equation, satisfied by the field *F*.

For the sake of mathematical rigor, one describes the formal exponentials of the intrinsic vector potential by symbols *V*(*ag*) that generate a *-algebra ${{\mathfrak {V}}}_0$, where $a \in {{\mathbb {R}}}$ and $g \in {{\mathcal {C}}}_1({{\mathbb {R}}}^4)$. They are subject to relations, expressing basic algebraic and locality properties of the potential, given by

2.1 $$\begin{aligned}&V(a_1 g) V(a_2 g) = V((a_1 + a_2) g) \, , \ V(g)^* = V(-g) \, , \ V(0) = {{\mathbf {1}}} \, , \ \ \end{aligned}$$

2.2 $$\begin{aligned}&V(\delta f_1) V(\delta f_2) = V(\delta f_1 + \delta f_2) \ \ \text {if} \ \ {\mathrm {supp}}\, f_1 \perp {\mathrm {supp}}\, f_2 \, , \end{aligned}$$

2.3 $$\begin{aligned}&\lfloor V(g_1) , V(g_2) \rfloor \, \in {{\mathfrak {V}}}_0 \cap {{\mathfrak {V}}}_0' \ \ \text {if} \ \ {\mathrm {supp}}\, g_1 \perp {\mathrm {supp}}\, g_2 \, . \end{aligned}$$

Here the symbol $\perp$ between two regions indicates that they are spacelike separated, ${{\mathfrak {V}}}_0 \cap {{\mathfrak {V}}}_0'$ denotes the center of ${{\mathfrak {V}}}_0$, and $\lfloor X_1, X_2 \rfloor \doteq X_1 X_2 X_1^* X_2^*$ is the group theoretic commutator.

The first relation (2.1) expresses basic properties of the exponential function and the fact that the generating operators are unitary. Relations (2.1) and (2.2) imply that the electromagnetic field is homogeneous, additive for functions having spacelike separated supports, and local. Finally, relation (2.3) embodies the information that the commutator of intrinsic vector potentials, integrated with spacelike separated test functions, lies in the center of the algebra, a fact verified in full generality in [3].

Let us mention as an aside that the preceding relations are a straightened version of corresponding (equivalent) relations presented in [3]. Examples of potentials which are nonlinear with regard to the test functions but comply with these conditions were presented in [4]; they have specific topological properties.

As is shown in [3], there exist faithful states on the algebra ${{\mathfrak {V}}}_0$. The corresponding GNS-representations determine C*-norms on this algebra. Proceeding to the completion of ${{\mathfrak {V}}}_0$ with regard to its maximal C*-norm, one arrives at a C*-algebra ${{\mathfrak {V}}}$, the universal algebra of the electromagnetic field. This algebra admits an automorphic action of the proper orthochronous Poincaré group which is fixed by the relations

2.4 $$\begin{aligned} \alpha _P(V(g)) \doteq V(g_P) \, , \quad P \in {{\mathcal {L}}}_+^\uparrow \ltimes {{\mathbb {R}}}^4 \, , \ g \in {{\mathcal {C}}}_1({{\mathbb {R}}}^4) \, , \end{aligned}$$

where $$x \mapsto g_P{}^\mu (x) \doteq L^\mu {}_\nu \, g^\nu (L^{-1}(x - y)) \in {{\mathcal {C}}}_1({{\mathbb {R}}}^4)$$ for $$P = (L,y)$$.

We shall show now that the algebra ${{\mathfrak {V}}}$ contains all ingredients for the analysis of electric charges. On one hand, it contains operators which determine these charges, on the other hand it allows for the action of automorphisms creating the corresponding fluxes. We recall these well-known facts by making use of the electromagnetic field *F* underlying our framework. In a second step we will recast the heuristic structures rigorously in terms of the universal algebra.

The electromagnetic field *F* determines the electric current *J* by the inhomogeneous Maxwell equation,

2.5 $$\begin{aligned} J(h) \doteq F(dh) = A_I(\delta d h) \, , \quad h \in {{\mathcal {D}}}_1({{\mathbb {R}}}^4) \, . \end{aligned}$$

Here ${{\mathcal {D}}}_1({{\mathbb {R}}}^4)$ is the space of real, vector-valued test functions with compact support and *dh* denotes the exterior derivative (the curl) of *h*. Noticing that $\delta d h \in {{\mathcal {C}}}_1({{\mathbb {R}}}^4)$, the second equality follows from the definition of the intrinsic vector potential. Choosing a Lorentz frame, the zero component of the current determines local charge operators for suitable choices of the test functions *h*. The value of these charges can be changed by maps of the intrinsic vector potential of the form

2.6 $$\begin{aligned} A_I(g) \mapsto A_I(g) + \varphi (g) {{\mathbf {1}}} \, , \quad g \in {{\mathcal {C}}}_1({{\mathbb {R}}}^4) \, , \end{aligned}$$

where $\varphi$ is a real linear functional on the space ${{\mathcal {C}}}_1({{\mathbb {R}}}^4)$. Since these maps are compatible with the linear properties and causal commutation relations of the intrinsic vector potential, they define automorphisms of the resulting algebra. Applying them to the current, one obtains

2.7 $$\begin{aligned} J(h) \mapsto J(h) + \varphi (\delta d h) {{\mathbf {1}}} \, , \quad h \in {{\mathcal {D}}}_1({{\mathbb {R}}}^4) \, . \end{aligned}$$

It reveals the fact that the charge can be changed by suitable choices of $\varphi$.

Turning to the details, we fix a Lorentz frame and a corresponding canonical coordinate system. Given any spacetime point $c = (c_0, {\varvec{c}})$, we consider the functions $x \mapsto h_c^\mu (x) \doteq \delta ^{\mu 0} \, \tau _{c_0}(x_0) \chi _{\varvec{c}}({\varvec{x}}) \in {{\mathcal {D}}}_1({{\mathbb {R}}}^4)$. Here $x_0 \mapsto \tau _{c_0}(x_0)$ is a test function with support in the interval $[c_0 - \varepsilon , c_0 + \varepsilon ]$, $\int \! dx_0 \, \tau _{c_0}(x_0) = 1$, and ${\varvec{x}}\mapsto \chi _{\varvec{c}}({\varvec{x}})$ is a smooth characteristic function which is equal to 1 in a given 3-ball of radius *r* around ${\varvec{c}}$ and 0 in the complement of a slightly larger ball with radius $r + \varepsilon$; the value of $\varepsilon > 0$ may be arbitrarily adjusted. The operator $J(h_c)$ describes a charge measurement in the 3-ball fixed by $\chi _{\varvec{c}}$ at the time fixed by $\tau _{c_0}$.

For the corresponding intrinsic vector potential $A_I(\delta d h_c)$, one obtains by a straightforward computation in the chosen coordinate system

2.8 $$\begin{aligned} x \mapsto \delta d h_c(x) = (\tau _{c_0}(x_0) \varvec{\Delta }\chi _{\varvec{c}}({\varvec{x}}), \, {\dot{\tau }}_{c_0}(x_0) \varvec{\nabla }\chi _{\varvec{c}}({\varvec{x}})) \, . \end{aligned}$$

Here $\varvec{\nabla }$ denotes the spatial gradient, $\varvec{\Delta }$ the Laplacian, and the dot $\dot{ }$ indicates a time derivative. Since the spatial derivatives of $\chi _{\varvec{c}}$ vanish in a 3-ball around ${\varvec{c}}$ and $\tau _{c_0}$ has support around $c_0$, the function $\delta d h_c$ has support in a cylindrical surface of height $2 \varepsilon$ and thickness $\varepsilon$ at distance *r* from *c*. This feature amounts to the known fact that charge measurements in a region can be replaced by flux measurements at its surface.

In the next step we exhibit linear maps $\varphi _m : {{\mathcal {C}}}_1({{\mathbb {R}}}^4) \rightarrow {{\mathbb {R}}}$, creating pairs of opposite charges which are localized at given spacelike separated regions around points $c_1, c_2 \in {{\mathbb {R}}}^4$. To this end we make use of a space $M({{\mathbb {R}}}^4)$ of vector-valued signed densities $\, m \,$ with compact support, satisfying the equation

2.9 $$\begin{aligned} \partial _\mu \, m^\mu (x) = q \big (\vartheta (c_1 - x) - \vartheta (c_2 - x) \big ) \, , \quad c_1 \perp c_2 \, ; \end{aligned}$$

here $\vartheta$ is a non-negative density with support around 0 which integrates to 1 and $\pm q \in {{\mathbb {R}}}$ are the values of the charges carried by the pair. Basic examples are the densities given by

2.10 $$\begin{aligned} x \mapsto m^\mu (x) \doteq q \, (c_1 - c_2)^\mu \! \int _0^1 \! du \, \vartheta (uc_2 + (1-u)c_1 -x) \, , \end{aligned}$$

which have support around the spacelike line connecting $c_1$ and $c_2$. The maps $\varphi _m$ are now defined as follows.

### Definition 2.1

Given $m \in M({{\mathbb {R}}}^4)$, the corresponding map $\varphi _m$ is defined by

2.11 $$\begin{aligned} \varphi _m(g) \doteq \int \! dx dy \ m^\mu (x) \, \Delta (x-y) \, g_\mu (y) \, , \quad g \in {{\mathcal {C}}}_1({{\mathbb {R}}}^4) \, , \end{aligned}$$

where $\Delta$ denotes the zero mass Pauli-Jordan commutator function. These expressions are well-defined since the convolutions of *g* and $\Delta$ yield smooth functions and the measures fixed by *m* have compact support. We shall refer to $\varphi _m$ as pair creating maps.

For the proof that the maps $\varphi _m$ have the desired properties, we apply them to the functions $h_c$ entering in the definition of local charge operators. Given $h_c$, let ${{\mathcal {O}}}(c)$ be the open double cone whose basis is a ball of radius $r - \varepsilon$ around ${\varvec{c}}$ in the time $c_0$-plane; it is the region where charges are measured. Similarly, let ${\overline{{{\mathcal {O}}}}}(c)$ be the closed double cone with basis of radius $r + 2 \varepsilon$ around ${\varvec{c}}$; it contains the support of $h_c$. That the resulting charge operator determines the charges carried by $\varphi _m$ is apparent from the subsequent lemma.

### Lemma 2.2

Let $\varphi _m$ be a pair creating map of charges in sufficiently small regions around spacelike separated points $c_1, c_2$ and let $h_c \in {{\mathcal {C}}}_1({{\mathbb {R}}}^4)$ be a function entering in the definition of local charge operators, as described above. Then

2.12 $$\begin{aligned} \varphi _m(\delta d h_c) = {\left\{ \begin{array}{ll} q &{} \text {if} \quad c_1 \in {{\mathcal {O}}}(c), \ c_2 \perp {\overline{{{\mathcal {O}}}}}(c) \\ -q &{} \text {if} \quad c_2 \in {{\mathcal {O}}}(c), \ c_1 \perp {\overline{{{\mathcal {O}}}}}(c) \\ 0 &{} \text {if} \quad c_1, c_2 \in {{\mathcal {O}}}(c) \ \ \text {or} \ \ c_1, c_2 \perp {\overline{{{\mathcal {O}}}}}(c) \, . \end{array}\right. } \end{aligned}$$

### Proof

Since the kernel fixed by the Pauli-Jordan function $\Delta$ is a bi-solution of the wave equation, one obtains on test functions the equality $\Delta \, \square = 0$, where $\square$ is the D’Alembertian. So one can replace the right action of the spatial Laplacian on $\Delta$ by a double time derivative. It yields by partial integration

2.13 $$\begin{aligned} \varphi _m(\delta d h_c)&= - \int \! dx dy \, m^\mu (x) \, {\dot{\Delta }}(x-y) \, \partial _\mu \tau _{c_0}(y_0) \chi _{\varvec{c}}({\varvec{y}}) \nonumber \\&= \int \! dx dy \, q \big (\vartheta (c_1 - x) - \vartheta (c_2 - x)\big ) \, {\dot{\Delta }}(x-y) \, \tau _{c_0}(y_0) \chi _{\varvec{c}}({\varvec{y}}) \, . \end{aligned}$$

Now the function

2.14 $$\begin{aligned} x \mapsto \int \! dy \, {\dot{\Delta }}(x - y) \tau _{c_0}(y_0) \chi _{\varvec{c}}({\varvec{y}}) \end{aligned}$$

is a smooth solution of the wave equation. It vanishes in the spacelike complement of ${\overline{{{\mathcal {O}}}}}(c)$ since $y \mapsto \tau _{c_0}(y_0) \chi _{\varvec{c}}({\varvec{y}})$ has support in that region. Moreover, in view of the specific choice of the latter function, the solution is equal to 1 in the double cone ${{\mathcal {O}}}(c)$, as is shown in the next paragraph. The statement of the lemma then follows immediately from the second line in relation (2.13), provided $\vartheta$ has support in a sufficiently small region around 0.

The remaining step is based on standard arguments, which we briefly recall. One first notices that the replacement of $\tau _{c_0}$ by another function $\tau _{c_0}^\prime$ with the same properties does not change the value of the resulting solution within the double cone ${{\mathcal {O}}}(c)$. This is so because the integral of $\tau _{c_0} - \tau _{c_0}^\prime$ vanishes, which implies $\tau _{c_0} - \tau _{c_0}^\prime = \partial _0 \sigma _{c_0}$, where $\sigma _{c_0}$ is a test function having also support in the interval $[c_0 - \varepsilon , c_0 + \varepsilon ]$. Moving the time derivative by a partial integration to ${\dot{\Delta }}$, replacing the resulting double derivative by the spatial Laplacian and moving the latter by partial integrations to $\chi _{\varvec{c}}$, one obtains for the difference between the two solutions the function

2.15 $$\begin{aligned} x \mapsto \int \! dy \, \Delta (x - y) \, \sigma _{c_0}(y_0) \varvec{\Delta }\chi _{\varvec{c}}({\varvec{y}}) \, . \end{aligned}$$

Now $y \mapsto \sigma _{c_0}(y_0) \varvec{\Delta }\chi _{\varvec{c}}({\varvec{y}})$ has support in a broadened cylindrical surface of height $2 \varepsilon$ at spatial distance *r* from *c*. Taking into account the support properties of $\Delta$, reflecting Huygens’ principle, it follows that the function (2.15) vanishes in ${{\mathcal {O}}}(c)$, as claimed.

In order to determine the values in ${{\mathcal {O}}}(c)$ of the solution of the wave Eq. (2.14), we may now replace the function $\tau _{c_0}$ by the Dirac delta function at $c_0$. Making use of the standard properties of ${\dot{\Delta }}$ in context of the Cauchy problem, we note that the resulting solution has, at time $c_0$, the value 1 in a ball of radius *r* around ${\varvec{c}}$ and its time derivative vanishes there. Thus, by the uniqueness properties of solutions of the Cauchy problem, it is equal to 1 in the double cone with this base. The original solution (2.14) therefore has the value 1 in the slightly smaller double cone ${{\mathcal {O}}}(c)$, completing the proof. $\square$

We turn now to the algebraic consequences of these observations. For the definition of pair creating automorphisms, we revert to the group of unitaries ${{\mathfrak {G}}}_0$, generated by the symbols *V*(*g*) with $g \in {{\mathcal {C}}}_1({{\mathbb {R}}}^4)$, which satisfy relations (2.1) to (2.3). From there we proceed to the trivial central extension ${{\mathbb {T}}}\times {{\mathfrak {G}}}_0$ of ${{\mathfrak {G}}}_0$ by the circle group ${{\mathbb {T}}}$. It is isomorphic to the unitary group in ${{\mathfrak {V}}}_0$ that consists of the elements $\eta \, V = V \eta$ with $\eta \in {{\mathbb {T}}}$ and $V \in {{\mathfrak {G}}}_0$. Its generating elements $\eta \, V(g)$ satisfy obvious extended equations, corresponding to relations (2.1) to (2.3).

Now, given any real linear map $\varphi : {{\mathcal {C}}}_1({{\mathbb {R}}}^4) \rightarrow {{\mathbb {R}}}$, we define a corresponding map $\beta _\varphi$ on the generating elements, putting

2.16 $$\begin{aligned} \beta _{\varphi }(\eta \, V(g)) \doteq \eta \, e^{i \varphi (g)} \, V(g) \, , \quad \eta \in {{\mathbb {T}}}\, , \, g \in {{\mathcal {C}}}_1({{\mathbb {R}}}^4) \, . \end{aligned}$$

In particular, $\beta _{\varphi }(\eta \, V(g)) = \eta \, \beta _{\varphi }(V(g))$ and $\beta _{\varphi }(V(g))^* = \beta _{\varphi }(V(g)^*)$. Since $\varphi$ is real and linear, it follows after a moment’s reflection that these maps are compatible with relations (2.1) to (2.3) and hence define morphisms, mapping the central extension onto itself. Regarding the elements of ${{\mathfrak {G}}}_0$ as basis of some complex vector space, we extend $\beta _\varphi$ linearly to that space. By the distributive law, we thereby obtain an automorphism of the *-algebra ${{\mathfrak {V}}}_0$. Since ${{\mathfrak {V}}}_0$ has been equipped with the maximal C*-norm, we conclude that $\beta _\varphi$ extends by continuity to an automorphism of the C*-algebra ${{\mathfrak {V}}}$. One also checks that these automorphisms satisfy under the action of Poincaré transformations *P* the equality $\alpha _P \beta _\varphi = \beta _{\varphi _P} \alpha _P$, where $\varphi _P(g) = \varphi (g_{P^{-1}})$ for $g \in {{\mathcal {C}}}_1({{\mathbb {R}}}^4)$.

We identify now the unitary exponential functions of the local charge operators with the symbols $V(\delta d h_c)$, where $\delta d h_c$ was given in relation (2.8). Picking $h_c$ and a pair creating map $\varphi _m$ as in Lemma 2.2, we obtain for the action of the corresponding automorphism $\beta _{m} \doteq \beta _{\varphi _m}$ on the exponentials

2.17 $$\begin{aligned} \beta _{m}(V(\delta d h_c)) = V(\delta d h_c) \cdot {\left\{ \begin{array}{ll} e^{iq} \! \! \! \! \! &{} \text {if} \ c_1 \in {{\mathcal {O}}}(c) \, , \ c_2 \perp {\overline{{{\mathcal {O}}}}}(c) \\ e^{-iq} \! \! \! \! \! &{} \text {if} \ c_2 \in {{\mathcal {O}}}(c) \, , \ c_1 \perp {\overline{{{\mathcal {O}}}}}(c) \\ 1 \! \! \! \! \! &{} \text {if} \ c_1, c_2 \in {{\mathcal {O}}}(c) \ \, \text {or} \ c_1, c_2 \perp {\overline{{{\mathcal {O}}}}}(c) \, . \end{array}\right. } \end{aligned}$$

These relations imply that the maps $\beta _{m}$ define outer automorphisms of ${{\mathfrak {V}}}$ if $q \ne 0$. In fact, any automorphism of ${{\mathfrak {V}}}$ that satisfies relation (2.17) is an outer automorphism. This assertion follows from the existence of a vacuum representation of ${{\mathfrak {V}}}$, describing the non-interacting electromagnetic field [3], where all unitaries $V(\delta d h_c)$ are represented by ${{\mathbf {1}}}$. Since these unitaries incorporate the local charge operators, relation (2.17) excludes the existence of unitary operators in ${{\mathfrak {V}}}$ which implement the action of $\beta _{m}$. In order to be able to implement it, one must extend the algebra ${{\mathfrak {V}}}$, as will be discussed in the subsequent section.

## Extensions of the universal algebra

The construction of an algebra, containing unitary elements implementing the action of $\beta _{m}$ on ${{\mathfrak {V}}}$, is based on group theoretic arguments, similar to those leading to Eq. (2.16). We proceed from the group ${{\mathfrak {H}}}_0$ that is generated by elements *W*(*m*), where $m \in M({{\mathbb {R}}}^4)$ are real densities with compact support, cf. Eq. (2.10). These generating elements are subject to the relations, $a_1, a_2 \in {{\mathbb {R}}}$,

3.1 $$\begin{aligned}&W(a_1 m) W(a_2 m) = W((a_1 + a_2) m) \, , \ W(m)^* = W(-m) \, , \ W(0) = {{\mathbf {1}}} \, , \quad \end{aligned}$$

3.2 $$\begin{aligned}&W(m_1) W(m_2) = W(m_1 + m_2) \ \ \text {if} \ \ {\mathrm {supp}}\, m_1 \perp {\mathrm {supp}}\, m_2 \, . \end{aligned}$$

They encode the information that the symbol *W*(*m*) has the algebraic properties of a unitary exponential function of some local generator with localization properties determined by the support of *m*. One can also consistently extend the action of the Poincaré transformations *P* to the group ${{\mathfrak {H}}}_0$, putting $\alpha _P(W(m)) = W(m_P)$, where $m_P$ is defined analogously to relation (2.4).

We then proceed to the semi-direct product ${{\mathfrak {H}}}_0 \ltimes ({{\mathbb {T}}}\times {{\mathfrak {G}}}_0)$, putting

3.3 $$\begin{aligned} W(m) V = \beta _{m}(V) W(m) \, , \quad W(m) \in {{\mathfrak {H}}}_0 \, , \ V \in ({{\mathbb {T}}}\times {{\mathfrak {G}}}_0) \, . \end{aligned}$$

The passage from the group of unitaries ${{\mathfrak {H}}}_0 \ltimes ({{\mathbb {T}}}\times {{\mathfrak {G}}}_0)$ to a *-algebra is now accomplished by standard arguments, cf. [3]. We extend ${{\mathfrak {H}}}_0 \ltimes ({{\mathbb {T}}}\times {{\mathfrak {G}}}_0)$ to a complex vector space ${{\mathfrak {W}}}_0$, choosing as its basis the elements *VW*, where $V \in {{\mathfrak {G}}}_0$ and $W \in {{\mathfrak {H}}}_0$. Making use of the distributive law and relation (3.3), one obtains an associative product on ${{\mathfrak {W}}}_0$. Adjoint operators are consistently defined in ${{\mathfrak {W}}}_0$ by canonically promoting to it the *-operation, acting on the group. In this manner ${{\mathfrak {W}}}_0$ becomes a *-algebra. One then defines a linear functional $\omega$ on ${{\mathfrak {W}}}_0$, putting

3.4 $$\begin{aligned} \omega (V W) = {\left\{ \begin{array}{ll} 0 &{} \text {if} \quad V W \ne {{\mathbf {1}}} \\ 1 &{} \text {if} \quad V W = {{\mathbf {1}}} \, . \end{array}\right. } \end{aligned}$$

Thus $\omega ((V_1 W_1)^* V_2 W_2) = \omega ( \beta (V^*_1 V_2) W_1^*W_2) = 0$ whenever $V_1 \ne V_2$ or $W_1 \ne W_2 \,$; here $\beta$ denotes the homomorphism induced by the adjoint action of $W_1$, mapping ${{\mathbb {T}}}\times {{\mathfrak {G}}}_0$ onto itself. It follows from this equality that $\omega$ defines a faithful state on ${{\mathfrak {W}}}_0$. Thus there exists a (maximal) C*-norm on ${{\mathfrak {W}}}_0$, so by completion it becomes a C*-algebra ${{\mathfrak {W}}}$.

Whereas the algebra ${{\mathfrak {W}}}$ contains the desired unitaries, implementing the automorphisms $\beta _m$ of ${{\mathfrak {V}}}$, its generating elements may not be regarded as observables. Because they admit non-trivial gauge transformations given by

3.5 $$\begin{aligned} \gamma _s(W(m)) = e^{i \int \! dx \, m^\mu (x) \partial _\mu s(x)} \, W(m) = e^{iq ((s *\vartheta )(c_2) - (s *\vartheta )(c_1)) } \, W(m) \, , \end{aligned}$$

where *s* is an arbitrary real scalar test function and the asterisk $*$ indicates convolution. Note that summands in *m* of elements in ${{\mathcal {C}}}_1({{\mathbb {R}}}^4)$ do not contribute here. It is also obvious that correspondent gauge transformations act trivially on the algebra ${{\mathfrak {V}}}$. By arguments already used, it is straightforward to prove that these transformations define automorphisms of the C*-algebra ${{\mathfrak {W}}}$.

In order to obtain gauge invariant unitaries which implement the action of $\beta _m$, we need to amend the framework by charged fields, describing the static matter. This is accomplished by elements of an abelian C*-algebra ${{\mathfrak {C}}}$ that is generated by all finite sums and products of unitary operators $\psi (\rho )$, where $\rho$ is a scalar density on ${{\mathbb {R}}}^4$ whose integral defines the charge carried by the operator. Furthermore, we assume that $\psi (\rho _1) \psi (\rho _2) = \psi (\rho _1 + \rho _2)$, $\psi (\rho )^* = \psi (-\rho )$, and $\psi (0) = \mathbf{1}$. Gauge transformations are defined on ${{\mathfrak {C}}}$, putting on its generating elements

3.6 $$\begin{aligned} \gamma _s(\psi (\rho )) = e^{i \int \! dx \, s(x) \rho (x) } \, \psi (\rho ) \, . \end{aligned}$$

Since ${{\mathfrak {C}}}$, being abelian, is a nuclear C*-algebra, the C*-tensor product ${{\mathfrak {W}}}\otimes {{\mathfrak {C}}}$ is uniquely defined. Its subalgebra of gauge invariant elements, denoted by $\underline{{{\mathfrak {W}}}\otimes {{\mathfrak {C}}}}$, contains ${{\mathfrak {V}}}$ and for any given $m \in M({{\mathbb {R}}}^4)$ the operators

3.7 $$\begin{aligned} {\underline{W}}(m) \doteq \psi (\vartheta _1) \, W(m) \ \psi (\vartheta _2)^* \, , \end{aligned}$$

where $\partial _\mu m^\mu (x) = q (\vartheta (c_1 - x) - \vartheta (c_2 - x))$ and $x \mapsto \vartheta _{1,2}(x) \doteq q \, \vartheta (c_{1,2} - x)$. These operators are gauge invariant as a consequence of relations (3.5) and (3.6). It is also apparent that they satisfy Eq. (3.3), *i.e. *their adjoint action implements the automorphism $\beta _m$ of ${{\mathfrak {V}}}$ as well. Thus the C*-algebra $\underline{{{\mathfrak {W}}}\otimes {{\mathfrak {C}}}}$ contains, apart from the electromagnetic field and corresponding local charge operators, also gauge invariant operators, creating pairs of opposite static charges and the corresponding fields.

## Representations

The algebra $\underline{{{\mathfrak {W}}}\otimes {{\mathfrak {C}}}}$, being a C*-algebra, has an abundance of representations. Yet since it describes static charges, it does not have representations where energy operators can be defined. We therefore restrict our attention to a subalgebra ${\underline{{{\mathfrak {W}}}}}$. It is generated by gauge invariant operators in ${{\mathfrak {W}}}$ built from elements of ${{\mathfrak {V}}}$ and operators *W*(*m*) with regular densities *m*. Such densities are obtained by choosing in relation (2.10) test functions $\vartheta$; we denote the corresponding regular subspace by ${\underline{M}}({{\mathbb {R}}}^4)$. Operators in ${\underline{{{\mathfrak {W}}}}}$ of the form

4.1 $$\begin{aligned} V_1 W(m_1) V_2 \cdots W(m_{n}) V_{n+1} \, , \qquad V_1, \dots V_{n+1} \in {{\mathfrak {V}}}\, , \end{aligned}$$

are gauge invariant if $\delta \, (m_1 + \cdots + m_n) = 0$. The regularity properties of *m* ensure that there exist positive energy representations of ${\underline{{{\mathfrak {W}}}}}$.

Our arguments are based on the Gupta-Bleuler formalism. Although we are dealing with gauge invariant operators, the Gupta-Bleuler fields are needed in order to obtain concrete representatives of the abstract unitaries *W*(*m*). They are not separately defined in these representations, but become meaningful in gauge invariant combinations. This fact provides an alternative argument that gauge bridges between static charges cannot be constructed by means of only the electromagnetic field.

We briefly sketch the well-known construction of the Gupta-Bleuler framework, cf. for example [12, 13]. Let ${{\mathcal {S}}}({{\mathbb {R}}}^4)$ be the space of real, vector-valued test functions. We denote the exponentials of the Gupta-Bleuler fields by the symbols

4.2 $$\begin{aligned} e^{iA(u)} \, , \quad u \in {{\mathcal {S}}}({{\mathbb {R}}}^4) \, . \end{aligned}$$

They satisfy the relations, $a \in {{\mathbb {R}}}$,

4.3 $$\begin{aligned} e^{iA(u)} e^{iaA(v)} = e^{i a \langle u, \Delta v \rangle } \, e^{iA(u + a v)} \, , \quad \big ( e^{iA(u)} \big ) {}^* = e^{- iA(u)} \, , \quad e^{iA(0)} = {\mathbf{1 }} \, , \end{aligned}$$

where we used the short hand notation

4.4 $$\begin{aligned} \langle u, \Delta v \rangle \doteq \int \! dx dy \, u^\mu (x) \, \Delta (x-y) \, v_\mu (y) \, . \end{aligned}$$

A linear, hermitian (but not positive), and Poincaré invariant functional $\varpi$ on the algebra generated by finite sums of these exponentials is given by

4.5 $$\begin{aligned} \varpi ( e^{iA(u)}) = e ^{\, (1/2) \, \langle u, \Delta _+ u \rangle } \, , \quad u \in {{\mathcal {S}}}({{\mathbb {R}}}^4) \, . \end{aligned}$$

Here $\Delta _+$ is the positive frequency part of the Pauli-Jordan function $\Delta$. The correlation functions

4.6 $$\begin{aligned} P \mapsto \varpi ( e^{iA(u)} \, e^{iA(v_P)} ) \, , \quad P \in {{\mathcal {L}}}_+^\uparrow \ltimes {{\mathbb {R}}}^4 \, , \ \ u,v \in {{\mathcal {S}}}({{\mathbb {R}}}^4) \, , \end{aligned}$$

are continuous and satisfy the relativistic spectrum condition.

One can identify now the generating elements of the abstract algebra ${{\mathfrak {W}}}$ with exponentials of the Gupta-Bleuler fields,

4.7 $$\begin{aligned} V(a g)&\mapsto e^{i a A(g)} \, , \quad g \in {{\mathcal {C}}}_1({{\mathbb {R}}}^4) \, , \ a \in {{\mathbb {R}}}\, , \nonumber \\ W(m)&\mapsto e^{i A(m)} \, , \quad m \in {\underline{M}}({{\mathbb {R}}}^4) \, , \ \delta \, m \ne 0 \, . \end{aligned}$$

As is easily checked on the basis of the relations (4.3), this identification complies with all defining relations of ${{\mathfrak {W}}}$. In particular, the automorphisms $\beta _m$, $m \in {\underline{M}}({{\mathbb {R}}}^4)$, on ${\underline{{{\mathfrak {W}}}}}$ are implemented by non-gauge invariant Gupta-Bleuler operators,

4.8 $$\begin{aligned} \beta _m({\underline{W}}) = e^{iA(m)} {\underline{W}} e^{-iA(m)} \, , \quad {\underline{W}} \in {\underline{{{\mathfrak {W}}}}} \, . \end{aligned}$$

The restriction of $\varpi$ to the gauge invariant subalgebra ${\underline{{{\mathfrak {W}}}}}$ is a positive functional, describing the vacuum state. This assertion follows from the fact that this restriction satisfies the Gupta-Bleuler condition, *w* being any scalar test function,

4.9 $$\begin{aligned} w \mapsto \varpi (e^{iA(u)} \, \delta A(w) \ e^{iA(v)} ) = 0 \quad \text {if} \quad \delta u = \delta v = 0 \, , \ \ u,v \in {{\mathcal {S}}}({{\mathbb {R}}}^4) \, . \end{aligned}$$

It is a consequence of relations (4.1) and (4.3). Proceeding to the GNS-representation induced by $\varpi \upharpoonright {\underline{{{\mathfrak {W}}}}}$, one obtains a continuous, unitary representation $P \mapsto U(P)$ of the Poincaré group, satisfying the relativistic spectrum condition. Since the restriction of $\varpi$ to ${\underline{{{\mathfrak {W}}}}}$ defines a pure state (as a consequence of its clustering properties), the operators *U*(*P*) are elements of the weak closure of ${\underline{{{\mathfrak {W}}}}}$. In this sense they are gauge invariant.

Let us likewise consider the representations of ${\underline{{{\mathfrak {W}}}}}$ which are induced by the pair creating automorphisms $\beta _m$, $m \in {\underline{M}}({{\mathbb {R}}}^4)$. There arises the question whether these representations are also covariant. Indeed, one obtains for the automorphic action ${\underline{W}} \mapsto {\underline{W}}_P$ of the Poincaré group on ${\underline{{{\mathfrak {W}}}}}$,

4.10 $$\begin{aligned} \beta _m({\underline{W}}_P) = e^{iA(m)} U(P) e^{-iA(m)} \, \beta _m({\underline{W}}) \ e^{iA(m)} U(P^{-1}) e^{-iA(m)} \, . \end{aligned}$$

But it would be premature to conclude from this observation that the (formally gauge invariant, unitary) operators $e^{iA(m)} U(P) e^{-iA(m)}$ are elements of the weak closure of ${\underline{{{\mathfrak {W}}}}}$, as would be necessary for an affirmative answer. Note that the representation *U* does not induce Poincaré transformations of the non-gauge invariant operators. Otherwise, the cocycles $P \mapsto e^{iA(m)} e^{-iA(m_P)}$ would be gauge invariant, which is obviously not the case. This feature contrasts with the case of covariant localizable charges, where the corresponding cocycles arising from spacetime translations in charged sectors are generically inner in the algebras of observables [9, Sect. II].

The analysis of these formal representations of spacetime transformations requires some detailed computations, which we briefly sketch. The action of the automorphisms $\beta _m$ on the field operators $F_{\mu \nu } = (\partial _\mu A_\nu - \partial _\nu A_\mu )$, being defined in the sense of operator-valued distributions, is given by

4.11 $$\begin{aligned} x \mapsto \beta _m(F_{\mu \nu }(x)) = F_{\mu \nu }(x) + (\partial _\mu \, {\underline{m}}_\nu - \partial _\nu \, {\underline{m}}_\mu )(x) \, {{\mathbf {1}}} \, . \end{aligned}$$

Here the zero mass shell restrictions ${\underline{m}}$ of the densities *m* enter,

4.12 $$\begin{aligned} x \mapsto {\underline{m}}_\mu (x) \doteq \int \! dy \, m_\mu (y) \, \Delta (y - x) \, . \end{aligned}$$

Since ${\underline{m}}_\mu$ is a solution of the wave equation, the representations induced by $\beta _m$ describe the electromagnetic field in presence of the classical currents $x \mapsto \partial _{\mu \,} \partial ^\nu {\underline{m}}_\nu (x)$. The generators of spacetime transformations in the vacuum representation are spatial integrals of normal ordered bilinear expressions, involving the electric and magnetic field. It follows that the generators in the representations induced by $\beta _m$ coincide with the generators in the vacuum representation, complemented by perturbations which are linear in the electromagnetic field, respectively constant. Since the functions ${\underline{m}}$ are smooth and have compact support at fixed times, these perturbations are well defined and the perturbed generators are hermitian operators. The proof that they are also selfadjoint requires some thorough analysis. One can show in this manner that the unitary time translations are affiliated with the weak closure of ${\underline{{{\mathfrak {W}}}}}$ and have generators which are bounded from below. We skip these computations and refer the interested reader to the literature on solutions of the quantized Maxwell equations involving external currents, cf. for example [7, Sect. V.B.3]. Underlying functional analytic details are discussed in [8].

## Conclusions

In the present article we have continued our analysis of the universal algebra of the electromagnetic field by discussing the effects of the presence of electric charges. In order to simplify the analysis, we have restricted our attention to static (infinitely heavy) charges. We have also avoided the discussion of infrared problems by considering only neutral pairs of charges which are localized at finite spatial distances. These restrictions allowed us to focus on the impact of the electric charges on the electromagnetic field.

The upshot of the present investigation is the insight that, as a consequence of Gauss’ law, the modifications of the electromagnetic field caused by electric charges cannot be described by operations involving only the electromagnetic field. Within our framework, it found its expression in the fact that the gauge bridges between charges induce outer automorphisms of the universal algebra. We have therefore enlarged this algebra to a bigger C*-algebra, containing localized unitary operators implementing these automorphisms. Noticing that these unitaries are not gauge invariant, we have added charged field operators, describing the static matter. Their combination with the unitaries inducing gauge bridges leads to well localized gauge invariant operators, which describe within the C*-algebraic framework bi-localized static charges.

By making use of the Gupta-Bleuler formalism, we have seen that the adjoint action of the unitary operators creating gauge bridges can be represented by exponential functions of non-observable gauge fields. In this manner gauge fields make their appearance within the framework of the gauge invariant universal algebra. The adjoint action of these non-observable unitaries also leads to physically meaningful representation of the universal algebra, in accordance with the empirical fact that the presence of electric charges has no adverse effects on the energetic properties of the electromagnetic field. Since we had modeled the charged matter as being static it was, however, meaningless to discuss its energetic properties as well.

We conclude this article with some remarks on recent related work by Mund, Rehren and Schroer [10, 11]. These authors recognized that one may subsume the additional degrees of freedom, which are inherent in the gauge bridges, into a scalar, non-local field, called escort field. Instead of regarding this escort as companion of the electromagnetic field, they propose to add it to the charged matter part. In this manner they produce gauge invariant but non-local field operators. Their approach may have computational advantages when considering dynamical matter since one can work from the outset in Hilbert space representations.

The views advocated by these authors are not in conflict with the present results. In contrast to their approach, we have put forward the localization properties of physical operations, such as the creation of charged pairs, without alluding from the outset to the idea of pushing compensating charges to infinity. Having worked with C*-algebras, we have avoided the usage of indefinite metric formalisms as well. Our excursion to the Gupta-Bleuler formalism was conducted merely for the sake of illustration. Thus in view of recent progress in the formulation of dynamical C*-algebras, cf. [1, 2] and references quoted there, one may hope that the algebraic framework can be expanded into a consistent theory for describing the electromagnetic field also in presence of dynamical charged matter.

## Data Availability

Data sharing is not applicable to this article as no new data were created or analyzed in this study.
